# Population-based surveillance for hypertension awareness, treatment, and control in nine districts - India Hypertension Control Initiative, 2018–19

**DOI:** 10.1038/s41371-025-01005-9

**Published:** 2025-03-22

**Authors:** Prabhdeep Kaur, Kamaraj Pattabi, Amirthammal Gunasekaran, Vettrichelvan Venkatasamy, Azhagendran Sivalingam, Sabarinathan Ramasamy, Sailaja Bitragunta, Tapas Chakma, Sampada D. Bangar, Meenakshi Sharma, Abhishek Kunwar, Kiran Durgad, Anupam K. Pathni, Sandeep S. Gill, Padmaja Jogewar, Madhavi Mallela, Ashish Saxsena, Bipin Gopal, Bidisha Das, Vishwajit Bharadwaj, Pooja Gaigaware, Sreedhar Chintala, Chakshu Joshi, Rupali Bharadwaj, Suyesh Shrivastava, Pankaj Uike, Yannick P. Puthussery, Gopinath T Sambandam, RS Dhaliwal, Balram Bhargava

**Affiliations:** 1 ICMR-National Institute of Epidemiology, Chennai, India; 2 ICMR-National Institute of Research in Tribal Health, Jabalpur, India; 3 ICMR-National AIDS Research Institute, Pune, India; 4 Indian Council of Medical Research, New Delhi, India; 5 Dept of Noncommunicable Diseases, WHO Country Office for India, New Delhi, India; 6 Resolve to Save Lives, New Delhi, India; 7 State NCD Cell, Department of Health and Family Welfare, Government of Punjab, Chandigarh, India; 8 State NCD Cell, Directorate of Health Services, Government of Maharashtra, Mumbai, India; 9 State NCD Cell, Department of Health, Medical and Family Welfare, Government of Telangana, Hyderabad, India; 10 State NCD Cell, Directorate of Health Services, Government of Madhya Pradesh, Bhopal, India; 11 State NCD Cell, Directorate of Health Services, Government of Kerala, Thiruvananthapuram, India; 12 IHCI Project, District NCD Cell, WHO-India, Bhatinda, Punjab India; 13 IHCI Project, District NCD Cell, WHO-India, Wardha, Maharashtra India; 14 IHCI Project, District NCD Cell, WHO-India, Karimnagar, Telangana India; 15 IHCI Project, District NCD Cell, WHO-India, Ratlam, Madhya Pradesh India; 16 IHCI Project, District NCD Cell, WHO-India, Chhindwara, Madhya Pradesh India; 17 IHCI Project, District NCD Cell, WHO-India, Thrissur, Kerala India; 18 IHCI Project, District NCD Cell, WHO-India, Kannur, Kerala India

**Keywords:** Diseases, Hypertension

## Abstract

Hypertension control is the crucial indicator for cardiovascular disease programs. We conducted a baseline cross-sectional survey to estimate hypertension awareness, treatment, and control in the selected districts in 2018–19, where the India Hypertension Control Initiative is being implemented. We conducted cross-sectional surveys in nine project districts for 18–69 years age group. The sample size was 624 per district. The study population was individuals with raised BP/diagnosed HT. We estimated the proportion and 95% confidence intervals (CI) for each district’s awareness, treatment, and control. We computed unadjusted and adjusted prevalence ratios (APR) with 95% CI for factors associated with BP control. Hypertension was defined as systolic blood pressure (SBP) > = 140 or diastolic blood pressure (DBP) > = 90 mmHg or treatment in the previous two weeks. Control was defined as SBP < 140 and DBP < 90 mmHg. Among 7047 who had hypertension, 52.4% were aware, 40.8% were on treatment, and 14.5% had BP control. BP control was below 5% in two districts, 5–15% in three districts, and more than 15% in four districts. Among hypertensives aware of the diagnosis, the factors (APR with 95% CI) associated with control were lack of alcohol consumption [1.28 (1.09–1.52)], recent visit to government [1.98 (1.57–2.50)] or private facility [1.99 (1.61–2.46)] and treatment with single drug [2.40 (1.98–2.90)] or multiple drugs [2.84 (2.27–3.55)]. The simple, rapid population-based surveys can document awareness, treatment, and control changes. Improving access to treatment for hypertension through the public or private sector should be a high priority for India.

## Background

Globally, of the 1.39 billion people with hypertension in 2010, nearly a billion lived in low and middle-income countries [1]. Uncontrolled blood pressure is a major risk factor for CVD morbidity and mortality. The global monitoring framework for NCDs includes reducing the prevalence of raised blood pressure by 25% by 2025 [2]. The India Hypertension Control Initiative (IHCI) is a multi-partner collaborative project that aims to accelerate this ambitious goal by strengthening hypertension management at the primary health care level in 100+ districts. IHCI interventions include standard hypertension treatment protocols, improvement in the availability of protocol drugs, task sharing, information system to monitor patient cohorts for blood pressure control, and decentralized care closer to the patient’s home [3].

While there is substantial data on the hypertension burden and rate of hypertension control in India, estimates of hypertension control at the district level are limited. According to the WHO STEPS framework, a 2017–2018 national survey found that nearly one-third of the adults (28.5%) had Hypertension in India, and 12.3% of hypertensives had controlled blood pressure in 2017–18 as per the national survey according to WHO STEPS framework. However, that survey design did not consider state/district level estimates [4, 5]. Two national-level surveys reported hypertension burden at the national, state, and district level in 2012–14 and 2015–16. A cross-sectional national-level survey of 1.3 million adults above 18 years of age conducted in 2012–14 reported a 25.3% prevalence of raised blood pressure [6]. The sample size of hypertensives per district was inadequate to measure changes in awareness, treatment, and control over time. Subsequently, the National Family Health survey-4 conducted in 2015–16 estimated hypertension among adults aged 15–49 years. Among 731,864 participants included in the analysis, hypertension prevalence, awareness, treatment, and control were 18.0%, 44.7%, 13.3%, and 7.9%, respectively [7]. The survey did not include the 50+ years of age group and therefore had lower hypertension estimates than the earlier surveys.

The limitations of existing surveys prevent their use as the baseline for estimating the effectiveness of IHCI interventions at the district level. Therefore, we undertook district-level surveys in nine districts using a standardized methodology and adequate sample size to enable comparisons over time. The main objective was to estimate the hypertension awareness, treatment, and control among people with hypertension in nine IHCI districts in India from 2018–19. The secondary objective was to determine the factors associated with blood pressure control among respondents with hypertension.

## Methods

### Study design, setting, and population

We established population-based surveillance with repeat cross-sectional surveys to document awareness, treatment, and control trends over time. We conducted a baseline survey during 2018–19 and plan to do a follow-up resurvey in 2023 in the same districts. The survey included nine out of 25 districts where phase-1 of the India Hypertension Control Initiative program was implemented. Two districts in each of the four states, namely Punjab, Madhya Pradesh, Telangana, and Kerala, and one district in Maharashtra, were included in the survey. The study population included adults aged 18–69 with raised blood pressure or already diagnosed hypertension and currently treated for hypertension. We selected non-contiguous districts with a mix of the urban-rural population. Predominantly urban districts were excluded.

### Inclusion and exclusion criteria

Eligible households are structures with a shared kitchen, where family members with at least one individual aged 18–69 years have been residing for more than six months. Eligible individuals included those aged 18–69 years living in the selected household at the survey time. Household-level exclusion criteria included the inability to enumerate the members due to lack of availability, refusal, lack of competent or appropriate respondents to give information, or lack of any eligible members in the household. Individual-level exclusion criteria included lack of availability for the interview after three attempts, refusal to participate, reclassification as ineligible (based on age, period of stay, pregnancy status), and inability to provide consent at the time of the survey.

### Sample size and sampling design

**Fig. 1:**
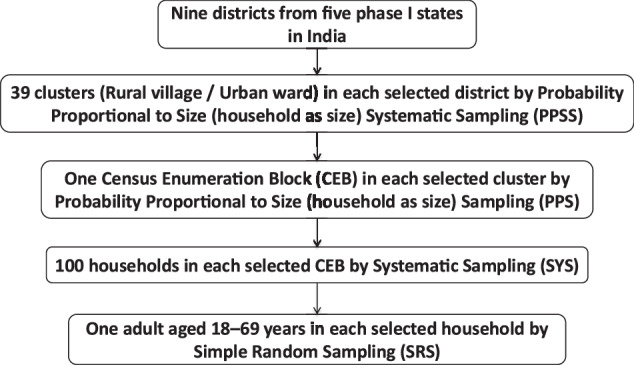
Sample size. Sample size for households and individuals for community-based survey in nine districts in India, 2018–19.

**Fig. 2:**
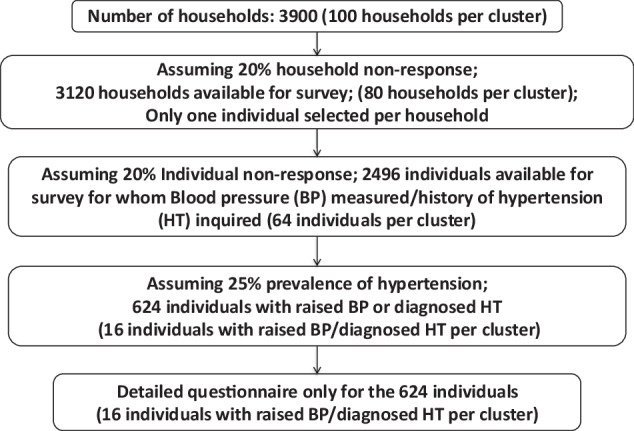
Sampling strategy. Stages of Sampling for community survey in nine districts in India, 2018–19.

The outcome was controlled blood pressure among the adults aged 18–69 years who had hypertension. Assuming blood pressure control of 20% at baseline and 30% at follow-up, intra-cluster correlation coefficient as 0.04, design effect of 1.6, with 95% confidence and a power of 90%, we required sample size of 624 adults with hypertension at baseline as well as for the follow-up resurvey. We computed a sample size of 624 adults aged 18–69 years with raised blood pressure (BP)/diagnosed Hypertension (HT) (Fig. 1). We assumed a 25% prevalence of hypertension; hence we aimed to survey four times the number of expected individuals with hypertension. We developed the sampling design to survey people with hypertension to reduce the cost and time per district. A multistage cluster sampling design was adopted for each study district (Fig. 2).

At the first stage, 39 clusters (villages/wards) were selected by probability proportional to size (household as size) systematic sampling from each district. One Census Enumeration Block (CEB) was selected from each of the selected clusters by probability proportional to size (household as size) sampling at the second stage. In the third stage, all the households in the CEB that met the eligibility criteria were enumerated. A systematic sampling selected a hundred households based on the total eligible households. We enumerated the ages 18–69 years with gender in the selected household at the fourth stage. We selected one adult aged 18–69 years by simple random sampling. Once an adult aged 18–69 years was randomly selected, we measured the blood pressure twice at five-minute intervals. We recorded the history of hypertension and treatment to determine the eligibility for the survey questionnaire and anthropometric measurements.

### Data collection

Overall data collection required approximately 90 days in each district with a team of a supervisor and six data collectors. Each cluster was completed in 2–3 days. The first day was to meet the village/ward leaders, mapping and enumerating the selected primary sampling unit. Most of the individual data collection was completed on the second day, and those unavailable on the second day were surveyed on the third day. The approximate survey cost per district was 22,000 USD.

Data was collected using Open Data Kit (ODK) based forms in Android Tablet. First, we visited the selected household based on the unique household ID of the CEB. The second step was the line listing of adults aged 18–69 years in the selected household with age and gender and then a random selection of one adult aged 18–69 years. The third step was to measure two blood pressure using a digital professional BP monitor (Omron 1300) [8]. We collected information regarding prior diagnosis and treatment of hypertension for the selected adult aged 18–69 years. If both readings were normal, no detailed data were collected; hence data collection for the adult aged 18–69 years with normal blood pressure took less than 10 min.

We collected a detailed questionnaire only among adults aged 18–69 years with raised systolic or diastolic blood pressure in the first or second reading or prior diagnosis and treatment of hypertension. The duration of data collection was approximately 25–30 min. The key variables included socio-demographic characteristics, behavioral risk factors, salt intake, treatment-related information, and counseling. Height and weight were measured using a stadiometer (SECA 213) and a weighing scale (SECA 803).

### Operational definitions

Current Smoker/smokeless tobacco user was defined as those who smoked/used smokeless tobacco either daily or occasionally at the time of the survey. Alcohol users included respondents who reported using alcohol 30 days prior to the survey. People who added salt before eating or reported eating salty foods were categorized as always/often, occasional, and never used. Eating meals outside the home was classified as people who ate at quick-serve locations always or often versus occasionally or never. The individuals were classified as aware of salt reduction if they considered reducing salt important or had an awareness that high salt cause health problems. Adequate physical activity included either vigorous activities at least 75 min per week or moderate activities at least 150 min per week. We classified the body mass index as per WHO recommendations [9].

Hypertension was defined based on the average of second and third readings. The criteria included systolic blood pressure (SBP) > = 140 or diastolic blood pressure (DBP) > = 90 mmHg or treatment with antihypertensive medications in the previous two weeks. Awareness of Hypertension was defined as individuals who reported being diagnosed by a health provider and satisfied the definition of hypertension mentioned above. The treatment category included people with hypertension who had taken medications in th**e** previous two weeks. Control was defined as SBP < 140 and DBP < 90 mmHg and taking the medication in the previous two weeks. Treatment providers were classified as informal, public, private, and AYUSH (Ayurveda, Yoga, Naturopathy, Unani, Siddha, and Homoeopathy) providers.

### Statistical analysis

We cleaned the data immediately following data collection and provided prompt feedback to the field teams. After adjusting for household non-response at cluster level and individual non-response at district level for each district, the appropriate sampling weights were calculated based on multistage sampling design. We projected the 18–69 years population for 2019 based on the 2011 census using the average exponential growth rate. Using the 2019 projected population of 18–69 years, calibrated sampling weight was calculated for each district’s adults aged 18–69 years. Complex sample weighted analysis was used to generate weighted frequencies and percentages and 95% confidence intervals for each district’s hypertension, awareness, treatment, and control outcome variables. We pooled data from all districts to analyse the factors associated with control among all and only among those aware of the hypertension status. We computed the mean (standard deviation) and median (Inter-quartile range) for age, body mass index, systolic and diastolic blood pressure. We calculated frequencies and proportions for all the categorical socio-demographic, behavioral, and treatment-related variables overall and by gender. We did a chi-square analysis to test the association between BP under control and all the key categorical variables.

We computed unadjusted Prevalence Ratio (PR) and 95% CI for each covariate for BP under control as an outcome using the Log-Binomial model. We used the hierarchical well-formulated model by including all the covariates for BP under control as a complete multivariate Log-Binomial model. Minus two log-likelihood ratio criteria were used to eliminate the covariates one by one based on non-significant highest *p*-value (least significance) and covariates with *p*-value > 0.20. However, the variables place of stay (rural/urban), gender and age group, and other covariates with *p*-value < = 0.20 were retained until the final reduced model. We presented the adjusted prevalence Ratio (APR) with 95% CI for each covariate for the full model and the final reduced model. All analyses were two-tailed, and a *P*-value of < 0.05 was considered statistically significant. We analyzed the data using the software STATA SE (version 17.0) (StataCorp LLC, Texas, USA).

### Human subjects protection

We obtained written informed consent from the respondents. The Institutional Ethics Committee approved the study. We used unique identifiers for the data collection and analysis.

## Results

We surveyed 29,227 adults 18–69 years, 7047 (24.1%) had hypertension. All the analyses are presented for the 7047 hypertensive individuals. The mean age was 51 years, and the mean BMI was 25.6 kg/m^2^. The mean age of newly detected hypertensives was 47 years compared to 54 years among participants already aware of the hypertension status.

The mean systolic blood pressure (SBP) was 152 mmHg, and the mean diastolic blood pressure (DBP) was 90 mmHg. The mean SBP was similar among males and females. The mean SBP (SD) was 150 (21) mmHg among people who were already aware compared to 154 (92) mmHg among people newly diagnosed with hypertension during the survey. The mean DBP (SD) was 87 (12) mmHg and 92 (10) respectively. The mean SBP (SD) was 148 (21) mmHg among those who were on treatment compared to 160 (19) mmHg among those who were aware but not on treatment. The mean DBP was 85 (12) mmHg and 94 (12) mmHg respectively.

**Fig. 3:**
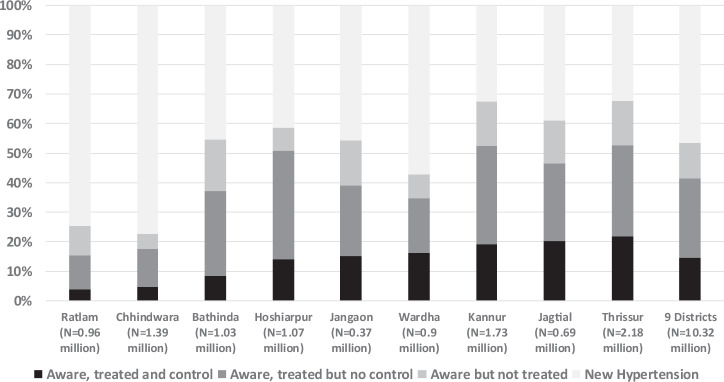
Hypertension care cascade. Cascade of hypertension awareness, treatment, and control in nine districts in India, 2018–19.

Among 7047 with hypertension, 52.4% were aware and 40.8% were on treatment. Of 7047, 52.6% (*n* = 3705) had SBP 140–159 mmHg or DBP 90–99 mmHg and 32.9% (*n* = 2319) had SBP > = 160 mmHg or DBP > = 100 mmHg. The blood pressure was under control for 14.5% (*n* = 1023) of participants. The treatment and control among aware were 77.8 and 27.7%, respectively. The weighted prevalence of hypertension ranged from 17.9% in Wardha (Maharashtra) to 33.0% in Hoshiarpur (Punjab) (Table 1). The proportion of hypertensives aware of the diagnosis was highest (67.0%) in the Kerala districts and lowest at 22.6% in Chhindwara (MP). The proportion aware was above 50% in six of the nine districts. The treatment coverage ranged from 52.9% in Thrissur (Kerala) to 15.3% in Ratlam (MP). Treatment coverage was above 50% in Hoshiarpur (Punjab) and Kerala districts. BP control was below 5% in two districts from MP, 5–15% in three districts, and above 15% in four districts (Table 1, Fig. 3). Treatment coverage and BP control was higher among females compared to males overall and in all the districts. Overall treatment coverage and BP control was higher among people above 45 years compared to the younger age group.

**Table 1:** Prevalence, awareness, treatment, and control of hypertension (weighted estimated) in nine districts in India, 2018–19.

District - State	Population 2019 (millions)	Hypertension Prevalence^a^	Aware of Hypertension Diagnosis^b^	Newly Detected Hypertension^c^	Treatment for Hypertension^d^	Blood pressure Under Control^e^	Uncontrolled HT among population^f^
		n (95%CI) in 1000	n (95%CI) in 1000	n (95%CI) in 1000	n (95%CI) in 1000	n (95%CI) in 1000	n in 1000
		% (95% CI)	% (95% CI)	% (95% CI)	% (95% CI)	% (95% CI)	%
**Hoshiarpur** - Punjab	1.07	354.8 (311.9–397.6)	208 (178.6–237.4)	146.7 (123.4–170)	180.8 (157–204.6)	49.9 (37.4–62.4)	*304.9*
		**33.0 (30.1–36.0)**	**58.6 (54.3–62.8)**	**41.4 (37.1–45.7)**	**51.0 (46.5–55.4)**	**14.1 (11.2–17.5)**	**28.4**
**Bathinda** - Punjab	1.03	295.6 (247.6–343.6)	161.6 (135.4–187.8)	134 (108.3–159.7)	109.4 (86.2–132.6)	25.1 (13.5–36.7)	*270.5*
		**28.7 (25.9–31.6)**	**54.7 (51.2–58.0)**	**45.3 (42.0–48.8)**	**37.0 (32.1–42.3)**	**8.5 (5.8–12.3)**	**26.2**
**Ratlam** - Madhya Pradesh	0.96	187.2 (158.8–215.6)	47.4 (35.4–59.5)	139.7 (115.9–163.5)	28.7 (20.5–36.8)	7.3 (3.1–11.5)	*179.9*
		**19.4 (17.5–21.5)**	**25.3 (20.4–31.0)**	**74.7 (68.9–79.6)**	**15.3 (11.9–19.4)**	**3.9 (2.2–6.8)**	**18.6**
**Chhindwara** - Madhya Pradesh	1.39	312.4 (263.7–361.1)	70.5 (44.9–96.2)	241.8 (207.4–276.2)	54.6 (34.8–74.5)	14.8 (7.9–21.7)	*297.6*
		**22.5 (20.3–24.9)**	**22.6 (17.1–29.3)**	**77.4 (70.7–82.9)**	**17.5 (13.1–23.0)**	**4.7 (3.2–7.0)**	**21.4**
**Wardha** - Maharastra	0.9	160.4 (133–187.8)	68.5 (49.9–87.2)	91.9 (77.5–106.3)	55.7 (40.7–70.8)	26.1 (17.3–35)	*134.3*
		**17.9 (15.6–20.4)**	**42.7 (36.5–49.2)**	**57.3 (50.8–63.5)**	**34.7 (29.4–40.6)**	**16.3 (12.5–21.0)**	**15.0**
**Jagtial** - Telangana	0.69	128.9 (113.6–144.2)	78.5 (65.6–91.3)	50.4 (43.4–57.5)	60.0 (48.7–71.4)	26 (20.3–31.7)	*102.9*
		**18.8 (16.8–20.9)**	**60.9 (55.9–65.6)**	**39.1 (34.4–44.1)**	**46.6 (41.7–51.5)**	**20.2 (16.9–24.0)**	**15.0**
**Jangaon** - Telangana	0.37	82.1 (70.4–93.8)	44.7 (37.2–52.1)	37.4 (30.6–44.2)	32 (26.9–37)	12.3 (9.6–15)	*69.8*
		**21.9 (20.1–23.9)**	**54.4 (49.4–59.3)**	**45.6 (40.7–50.6)**	**38.9 (34.2–43.9)**	**15.0 (12.3–18.1)**	**18.6**
**Kannur** - Kerala	1.73	384.9 (351.5–418.2)	259.3 (234.8–283.9)	125.5 (101.1–150)	202.4 (180.7–224)	73.7 (60.5–86.8)	*311.2*
		**22.2 (20.5–24.0)**	**67.4 (62.3–72.1)**	**32.6 (27.9–37.7)**	**52.6 (47.1–58.0)**	**19.1 (15.6–23.2)**	**18.0**
**Thrissur** - Kerala	2.18	573.7 (523.5–623.9)	388.6 (351.6–425.6)	185.1 (156.1–214.0)	303.4 (271.3–335.5)	125.9 (104.6–147.2)	*447.8*
		**26.3 (24.5–28.2)**	**67.7 (63.9–71.4)**	**32.3 (28.6–36.1)**	**52.9 (48.6–57.1)**	**21.9 (18.8–25.4)**	**20.5**
**5 States** - 9 Districts	10.32	2480.0 (2373.3–2586.7)	1327.2 (1259.2–1395.2)	1152.7 (1086.2–1219.3)	1027.1 (970.1–1084.1)	361.2 (329.0–393.4)	*2118.8*
		**24.0 (23.2–24.8)**	**53.5 (51.9–55.2)**	**46.5 (44.8–48.1)**	**41.4 (39.7–43.1)**	**14.6 (13.4–15.8)**	**20.5**

^a^SBP > = 140/DBP > = 90 mmHg based on average of 2nd & 3rd BP or treatment with antihypertensive medications in the previous two weeks.

^b^Reported being diagnosed by a health provider.

^c^Diagnosed during the survey with SBP > = 140/DBP > = 90 mmHg based on average of 2nd & 3rd BP.

^d^Treatment with antihypertensive medications in the previous two weeks.

^e^SBP < 140 and DBP < 90 mmHg and taking the antihypertensive medication in the previous two weeks.

^f^SBP > = 140/DBP > = 90 mmHg based on average of 2nd & 3rd BP.

We analyzed the factors associated with BP control among all hypertensives and the sub-group aware of the diagnosis. BP control was higher among females, the older age group, non-smokers/smokeless tobacco users, and non-users of alcohol (*p* < 0.05) compared to the reference categories (Table 2). Patients who knew or were counseled about salt reduction, had adequate physical activity, had one or more comorbidities, took treatment in Government or private facilities, and were on multiple drugs had higher control when compared to reference categories (*p* < 0.05) (Table 2). Among all hypertensives, the factors (Adjusted PR with 95% CI) associated with control were being female [1.14 (1.01–1.30)], non-user of smokeless tobacco [1.25 (1.03–1.52)], non-consumer of alcohol [1.25 (1.05–1.49)], recent treatment at Government [12.29 (9.56–15.82)] or private facility [11.69 (9.22–14.81)], counseled for tobacco use [1.28 (1.12–1.47)] and counseled for salt reduction [1.26 (1.07–1.48] (Table 3).

**Table 2:** Factors associated with blood pressure under control in nine districts in India, 2018–19.

Categories	Factors associated for BP Under Control among all people with hypertension (*n* = 7047)*				Factors associated for BP Under Control among people aware of hypertension diagnosis (*n* = 3691)				
	BP Under Control (*n* = 1023)		BP Not Under Control (*n* = 6024)		BP Under Control (*n* = 1023)		BP Not Under Control (*n* = 2668)		
	n	%	n	%	n	%	n	%	*p*-value
Area									
Rural	589	13.6	3736	86.4	589	28.2	1500	71.8	0.458
Urban	434	15.9	2288	84.1	434	27.1	1168	72.9	
Gender									
Male	303	10.2	2653	89.8	303	24.0	961	76.0	0.000
Female/Transgender	720	17.6	3371	82.4	720	29.7	1707	70.3	
Age Group (Years)									
18–44	160	8.1	1818	91.9	160	24.7	488	75.3	0.0221
45–59	427	15.3	2367	84.7	427	27.3	1135	72.7	
60–69	436	19.2	1839	80.8	436	29.4	1045	70.6	
Education									
No Formal Education	269	12.7	1852	87.3	269	26.2	758	73.8	0.199
Formal Education	754	15.3	4172	84.7	754	28.3	1910	71.7	
Occupation									
Professionals/Landowner/ Senior managerial	264	14.0	1619	86.0	264	26.9	718	73.1	0.225
Employed in non manual occupation	70	12.0	513	88.0	70	25.0	210	75.0	
Manual skilled or unskilled worker	169	9.8	1562	90.2	169	26.0	481	74.0	
Home Maker	520	18.2	2330	81.8	520	29.2	1259	70.8	
Current smoker									
Yes	54	6.6	760	93.4	54	20.0	216	80.0	0.003
No	969	15.5	5264	84.5	969	28.3	2452	71.7	
Current Smokeless tobacco user									
Yes	96	7.9	1115	92.1	96	23.8	308	76.2	0.060
No	927	15.9	4909	84.1	927	28.2	2360	71.8	
Alcohol use									
Yes	140	8.9	1433	91.1	140	21.1	524	78.9	0.000
No	883	16.1	4591	83.9	883	29.2	2144	70.8	
Adding salt before eating or eating high salt foods									
Often	185	10.0	1672	90.0	185	25.0	554	75.0	0.177
Occasional	668	15.6	3623	84.4	668	28.2	1698	71.8	
Never	170	18.9	729	81.1	170	29.0	416	71.0	
Eating meals outside home									
Frequently	125	11.2	988	88.8	125	26.6	344	73.4	0.582
Occasionally/never	898	15.1	5036	84.9	898	27.9	2324	72.1	
Aware regarding salt reduction									
No	160	6.6	2280	93.4	160	22.9	540	77.1	0.001
Yes	863	18.7	3744	81.3	863	28.8	2128	71.2	
Physical Activity									
Adequate	818	15.3	4515	84.7	818	28.8	2023	71.2	0.008
Inadequate/NotDone	205	12.0	1509	88.0	205	24.1	645	75.9	
Recent Facility Visit									
Public sector	220	34.0	427	66.0	220	34.0	427	66.0	0.000
Private sector	707	32.1	1498	67.9	707	32.1	1498	67.9	
AYUSH	7	19.4	29	80.6	7	19.4	29	80.6	
Informal providers/No treatment	89	2.1	4070	97.9	89	11.1	714	88.9	
Comorbidities									
One or more comorbidities	501	24.1	1579	75.9	501	29.6	1190	70.4	0.017
No Comorbidity	522	10.5	4445	89.5	522	26.1	1478	73.9	
Received counseling to stop tobacco use									
Yes	227	19.8	922	80.2	227	29.7	538	70.3	0.174
No	796	13.5	5102	86.5	796	27.2	2130	72.8	
Received counseling to reduce Salt									
Yes	862	23.6	2796	76.4	862	29.0	2106	71.0	0.000
No	161	4.7	3228	95.3	161	22.3	562	77.7	
BMI WHO (kg/m2)									
Under Weight (<18.50)	37	10.0	333	90.0	37	31.4	81	68.6	0.0145
Normal Weight (18.50–24.99)	403	13.1	2666	86.9	403	28.2	1028	71.8	
Over Weight (25.00–29.99)	413	17.0	2014	83.0	413	29.8	975	70.2	
Obese (> = 30.00)	170	14.4	1011	85.6	170	22.5	584	77.5	
Hypertension drugs									
Single	613	33.9	1194	66.1	613	33.9	1194	66.1	0.000
Combination	160	33.2	322	66.8	160	33.2	322	66.8	
Multiple	129	39.1	201	60.9	129	39.1	201	60.9	
No Drug	121	2.7	4307	97.3	121	11.3	951	88.7	

*p value < 0.05 for all variables.

**Table 3:** Prevalence Ratio for factors associated with blood pressure under control among all people with hypertension in nine districts in India, 2018–19 (*N* = 7047).

Categories	Unadjusted Prevalence Ratio (PR)	Adjusted PR (Model 1^a^)	Adjusted PR (Model 2^b^)
	Unadjusted PR (95% CI)	Adjusted PR (95% CI)	Adjusted PR (95% CI)
Area			
Rural	1.00 (Ref)	1.00 (Ref)	1.00 (Ref)
Urban	1.17 (1.04–1.31)	0.90 (0.80–1.00)	0.91 (0.82–1.01)
Gender			
Male	1.00 (Ref)	1.00 (Ref)	1.00 (Ref)
Female/Transgender	1.72 (1.51–1.95)	1.26 (1.08–1.48)	1.14 (1.01–1.30)
Age Group (Years)			
18–44	1.00 (Ref)	1.00 (Ref)	1.00 (Ref)
45–59	1.89 (1.59–2.24)	1.05 (0.90–1.23)	1.05 (0.90–1.22)
60–69	2.37 (2.00–2.81)	1.10 (0.94–1.30)	1.09 (0.93–1.27)
Education			
No Formal Education	1.00 (Ref)	1.00 (Ref)	
Formal Education	1.21 (1.06–1.37)	1.09 (0.95–1.25)	
Occupation			
Manual work	1.00 (Ref)	1.00 (Ref)	
Sedentary	1.44 (1.20–1.72)	1.00 (0.85–1.19)	
Employed	1.23 (0.95–1.60)	0.99 (0.79–1.26)	
Homemaker	1.87 (1.59–2.20)	0.88 (0.75–1.05)	
Current smoker			
Yes	1.00 (Ref)	1.00 (Ref)	1.00 (Ref)
No	2.34 (1.80–3.05)	1.26 (0.97–1.62)	1.28 (0.99–1.65)
Current Smokeless tobacco user			
Yes	1.00 (Ref)	1.00 (Ref)	1.00 (Ref)
No	2.00 (1.64–2.45)	1.23 (1.01–1.49)	1.25 (1.03–1.52)
Alcohol use			
Yes	1.00 (Ref)	1.00 (Ref)	1.00 (Ref)
No	1.81 (1.53–2.15)	1.26 (1.06–1.50)	1.25 (1.05–1.49)
Adding salt before eating or eating high salt foods			
Often	1.00 (Ref)	1.00 (Ref)	
Occasional	1.56 (1.34–1.82)	1.00 (0.87–1.14)	
Never	1.90 (1.56–2.30)	1.02 (0.86–1.21)	
Eating meals outside home			
Frequently	1.00 (Ref)	1.00 (Ref)	
Occasionally/never	1.35 (1.13–1.61)	0.94 (0.80–1.10)	
Aware regarding salt reduction			
No	1.00 (Ref)	1.00 (Ref)	1.00 (Ref)
Yes	2.86 (2.43–3.36)	1.11 (0.95–1.31)	1.14 (0.98–1.33)
Physical activity			
Inadequate/NotDone	1.00 (Ref)	1.00 (Ref)	
Adequate	1.28 (1.11–1.48)	1.07 (0.93–1.22)	
Recent Facility Visit			
Informal providers/No treatment	1.00 (Ref)	1.00 (Ref)	1.00 (Ref)
Public sector	15.89 (12.60–20.04)	12.15 (9.42–15.66)	12.29 (9.56–15.82)
Private sector	14.98 (12.09–18.56)	11.62 (9.15–14.75)	11.69 (9.22–14.81)
AYUSH	9.09 (4.53–18.22)	6.83 (3.38–13.78)	6.78 (3.36–13.67)
Co-Morbidities			
No Comorbidity	1.00 (Ref)	1.00 (Ref)	
One or more comorbidities	2.29 (2.05–2.56)	1.06 (0.96–1.18)	
Received counseling to stop tobacco use			
No	1.00 (Ref)	1.00 (Ref)	1.00 (Ref)
Yes	1.46 (1.28–1.67)	1.27 (1.10–1.46)	1.28 (1.12–1.47)
Received counseling to reduce Salt			
No	1.00 (Ref)	1.00 (Ref)	1.00 (Ref)
Yes	4.96 (4.22–5.83)	1.25 (1.06–1.47)	1.26 (1.07–1.48)
Body mass index (kg/m2)			
Normal Weight (18.50–24.99)	1.00 (Ref)	1.00 (Ref)	1.00 (Ref)
Under Weight (<18.50)	0.76 (0.55–1.05)	1.17 (0.90–1.54)	1.17 (0.89–1.53)
Over Weight (25.00–29.99)	1.29 (1.14–1.47)	1.02 (0.91–1.14)	1.03 (0.92–1.15)
Obese (> = 30.00)	1.10 (0.93–1.29)	0.75 (0.64–0.87)	0.75 (0.64–0.88)

^a^Model 1 - All variables listed in the table were included.

^b^Model 2 - Variables with *p*-value < 0.2 in model 1 were included in model 2.

The factors (Adjusted PR with 95% CI) associated with control were non-consumption of alcohol [1.28 (1.09–1.52)] recent visit to the Government [1.98 (1.57–2.50)] or private facility [1.99 (1.61–2.46)] and treatment with single drug [2.40 (1.98–2.90)] or multiple drugs [2.84 (2.27–3.55)] among hypertensives who were aware of the status (Table 4).

**Table 4:** Prevalence ratio for factors associated with blood pressure under control among people already aware of the hypertension diagnosis in nine districts in India, 2018–19 (*N* = 3691).

Categories	Unadjusted Prevalence Ratio (PR)	Adjusted PR (Model 1^a^)	Adjusted PR (Model 2^b^)
	Unadjusted PR (95% CI)	Adjusted PR (95% CI)	Adjusted PR (95% CI)
Area			
Rural	1.00 (Ref)	1.00 (Ref)	1.00 (Ref)
Urban	0.96 (0.86–1.07)	0.85 (0.77–0.95)	0.86 (0.78–0.96)
Gender			
Male	1.00 (Ref)	1.00 (Ref)	1.00 (Ref)
Female/Transgender	1.24 (1.10–1.39)	1.18 (1.01–1.38)	1.11 (0.99–1.26)
Age Group (Years)			
18–44	1.00 (Ref)	1.00 (Ref)	1.00 (Ref)
45–59	1.11 (0.95–1.29)	0.89 (0.77–1.03)	0.88 (0.76–1.02)
60–69	1.19 (1.02–1.39)	0.89 (0.76–1.04)	0.87 (0.75–1.01)
Education			
No Formal Education	1.00 (Ref)	1.00 (Ref)	
Formal Education	1.08 (0.96–1.22)	1.09 (0.95–1.25)	
Occupation			
Manual Work	1.00 (Ref)	1.00 (Ref)	
Sedentary	1.03 (0.88–1.22)	1.02 (0.86–1.19)	
Employed	0.96 (0.75–1.22)	0.98 (0.78–1.24)	
Homemaker	1.12 (0.97–1.30)	0.93 (0.79–1.10)	
Current smoker			
Yes	1.00 (Ref)	1.00 (Ref)	
No	1.42 (1.11–1.81)	1.15 (0.89–1.48)	
Current Smokeless tobacco user			
Yes	1.00 (Ref)	1.00 (Ref)	1.00 (Ref)
No	1.19 (0.99–1.42)	1.19 (0.98–1.44)	1.20 (0.99–1.44)
Alcohol use			
Yes	1.00 (Ref)	1.00 (Ref)	1.00 (Ref)
No	1.38 (1.18–1.62)	1.27 (1.07–1.51)	1.28 (1.09–1.52)
Adding salt before eating or eating high salt foods			
Often	1.00 (Ref)	1.00 (Ref)	
Occasional	1.13 (0.98–1.30)	0.99 (0.86–1.13)	
Never	1.16 (0.97–1.38)	0.98 (0.82–1.16)	
Eating meals outside home			
Frequently	1.00 (Ref)	1.00 (Ref)	
Occasionally/never	1.04 (0.89–1.23)	0.96 (0.82–1.13)	
Aware regarding salt reduction			
No	1.00 (Ref)	1.00 (Ref)	
Yes	1.26 (1.09–1.46)	0.97 (0.84–1.13)	
Physical activity			
Inadequate/NotDone	1.00 (Ref)	1.00 (Ref)	
Adequate	1.19 (1.04–1.36)	1.07 (0.94–1.22)	
Recent Facility Visit			
Informal providers/No treatment	1.00 (Ref)	1.00 (Ref)	1.00 (Ref)
Public sector	3.07 (2.45–3.83)	1.99 (1.58–2.52)	1.98 (1.57–2.50)
Private sector	2.89 (2.36–3.55)	1.99 (1.61–2.47)	1.99 (1.61–2.46)
AYUSH	1.75 (0.88–3.51)	1.11 (0.55–2.22)	1.11 (0.55–2.22)
Co-Morbidities			
No Comorbidity	1.00 (Ref)	1.00 (Ref)	
One or more comorbidities	1.13 (1.02–1.26)	0.98 (0.89–1.09)	
Received counseling to stop tobacco use			
No	1.00 (Ref)	1.00 (Ref)	1.00 (Ref)
Yes	1.09 (0.96–1.23)	1.18 (1.02–1.35)	1.15 (1.01–1.31)
Received counseling to reduce Salt			
No	1.00 (Ref)	1.00 (Ref)	
Yes	1.30 (1.12–1.51)	0.98 (0.85–1.13)	
Body mass index (kg/m2)			
Normal Weight (18.50–24.99)	1.00 (Ref)	1.00 (Ref)	1.00 (Ref)
Under Weight (<18.50)	1.11 (0.84–1.47)	1.16 (0.90–1.51)	1.17 (0.90–1.51)
Over Weight (25.00–29.99)	1.06 (0.94–1.19)	0.99 (0.89–1.11)	1.01 (0.90–1.12)
Obese (> = 30.00)	0.80 (0.68–0.93)	0.74 (0.64–0.86)	0.75 (0.64–0.87)
Drug			
No Drug	1.00 (Ref)	1.00 (Ref)	1.00 (Ref)
Single	3.00 (2.51–3.60)	2.37 (1.95–2.87)	2.40 (1.98–2.90)
Combination	2.94 (2.38–3.63)	2.39 (1.92–2.98)	2.37 (1.91–2.95)
Multiple	3.46 (2.79–4.29)	2.81 (2.24–3.53)	2.84 (2.27–3.55)

^a^Model 1 - All variables listed in the table were included.

^b^Model 2 - Variables with *p*-value < 0.2 in model 1 were included in model 2.

## Discussion

We developed a rapid survey methodology for population-based surveillance of hypertension control which documented high variations in treatment coverage and control. The repeat surveys can be implemented periodically with limited resources at the district level to estimate trends in awareness, treatment, and control of hypertension over time.

Hypertension prevalence, treatment, and control were variable across the study districts at different levels of epidemiological transition. The Global Burden of Disease Study documented early transition and high burden of CVD mortality in states such as Kerala and Punjab [10]. Several states have initiated state-level STEPS surveys, although it is not routine practice in all states. The high prevalence of hypertension in districts from Punjab and Kerala was consistent with the state-level STEPS surveys from these states [11]. Overall, treatment coverage was 16%, and only 12.3% of the hypertensives had controlled BP in 2017–18 in India [12]. Since various districts have very different baseline levels, national-level estimates might not be helpful to document the effect of district-level interventions in improving awareness, treatment, and control. State-level estimates were available for two (Punjab, Kerala) of the five states included in the study. Overall, nearly one-third of the hypertensives were on treatment in Punjab (30%) and Kerala (36%) as per STEPS surveys in 2014–15 and 2016–17, respectively [11, 13, 14]. We observed higher treatment coverage in the surveyed districts in Kerala and one district in Punjab (Hoshiarpur) than state-level STEPS survey estimates. The difference could be due to high variations across districts or improvement in the treatment coverage during the time period between the state STEPS surveys and our survey. In addition, BP control in the surveyed districts was also higher than the overall control for Kerala (12.4%) STEPS survey, possibly due to improvement in the treatment coverage [13].

We examined the role of socio-demographic factors for BP control. Control was higher among older adults and women, consistent with studies from India and globally. Control was 28.8% in an extensive survey among 64,427 adults above 45 years in 2017–18 in India. Control was lower among males compared to females [15]. The PURE study with participants from 17 countries, including LMIC, reported higher BP control among women [16]. Similar findings were documented in a state-level STEPS survey from Punjab, India [11]. Contrary to other studies, we did not document higher control among more educated participants [15, 16]. Among the behavioral factors, BP control was poor among alcohol users. Reducing alcohol use improves BP control among heavy drinkers [17]. Healthcare workers can be trained to counsel for reducing alcohol use in primary care settings. One of the encouraging findings was better control among patients counseled for salt reduction and who took antihypertensives. Scaling the treatment with antihypertensives and salt reduction strategies should be given high priority to address the poor treatment coverage (16%) in India [12].

WHO STEPS survey recommends a standard methodology to estimate the prevalence and trends for eight NCD risk factors; however, the surveys are expensive and time-consuming [5]. Moreover, the surveys are representative at the national or state level but do not enable district-level comparisons over time [4, 11, 13]. Recent National Family Health Survey-5, which primarily focuses on reproductive and child health indicators, did include BP measurement. However, the sampling methodology included all adults above 18 years in the selected households, influencing the key indicators. In addition, sample size at the district level may not be adequate to measure the change in BP control over time. IHCI project interventions aim to increase BP control by improving access to affordable patient-friendly services at the primary care level in 100+ districts [3]. The intervention is unlikely to change the other NCD risk factors in the population. Therefore we did not estimate the prevalence of risk factors for the whole population. Moreover, the prevalence of NCD risk factors is also available from other surveys [4, 6]. We limited the detailed survey to people with raised blood pressure during a survey or treated for hypertension to reduce the fieldwork and survey duration. While designing the study, we included the best practices, WHO STEPS, and Global Adult Tobacco Survey (GATS). GATS is a global standard for systematically monitoring adult tobacco use and tracking key control indicators. We incorporated sample design features and recommendations related to quality assurance procedures to ensure sufficient statistical quality. These included incorporating household and individual non-response adjustments to the cluster and stratum level weighting calculation and applying sampling weights and post-stratification adjustments. Additionally, we reviewed patterns of person-level refusal rates, item non-response, and the multiplicative effect of variance estimation, which resulted in a well-designed survey and standardized weights [18].

The study’s strength was survey methodology which used the best practices but reduced the overall fieldwork, duration, and cost. We computed the sample size to ensure an adequate number of people with raised blood pressure or treated hypertension to compare proportions during repeat surveys. One of the limitations of our methodology is the lack of inclusion of non-intervention districts to enable comparisons in change for various indicators. However, the implementation in different states is influenced by socioeconomic development, the population literacy level, availability of human resources, drugs, and involvement of community health workers. Hence, we will be able to compare the outcomes over time in the context of variations in the level of implementation. Another limitation was using the average second and third reading in the same visit to classify the individuals with raised blood pressure. This was aligned to the best practice for identifying people with raised blood pressure during surveys; however the diagnosis of hypertension should be as per the treatment guidelines, where the blood pressure measurement on different days is recommended.

We established population-based surveillance for hypertension awareness, treatment, and control indicators in nine districts where a focused primary care intervention is ongoing to improve BP control. Our methodology may be helpful in similar settings where hypertension interventions are implemented at the sub-national level. Over time, the extent of change in the key hypertension indicators will guide a need to modify or change the intervention strategies.

## Summary table

### What is known about this topic

- In India, only 12.3% of people with hypertension had blood pressure under control in 2017–18 using the WHO STEPS survey methodology.
- In India, the level of implementation of hypertension interventions is highly variable across districts. Hence, district-level hypertension indicators namely awareness, treatment and control will be useful to track the progress of hypertension control.

### What this study adds

- We developed a rapid survey methodology to estimate the hypertension indicators in districts where a primary care intervention was ongoing.
- There was a high variation in district-level hypertension control in nine study districts. BP control was below 5% in two districts, 5–15% in three districts, and above 15% in four districts.
- The methodology may be helpful in similar settings to track the progress of hypertension control at the sub-national level.

## Supplementary information

Supplementary Tables

## Data Availability

Data are available from the corresponding author at request.
