# Supplementary material for: Population-based surveillance for hypertension awareness, treatment, and control in nine districts - India Hypertension Control Initiative, 2018–19
Source: J Hum Hypertens. 2025 Mar 22;39(5):376–86. doi: 10.1038/s41371-025-01005-9 (PMC12069080; doi:10.1038/s41371-025-01005-9)
Supplement: Supplementary file 1 — Supplementary Tables [file 41371_2025_1005_MOESM1_ESM.pdf]

Supplementary Table 1: List of IHCI districts and selected districts for the survey

States	IHCI project districts	Community survey districts
Punjab	Mansa, Bhatinda, Gurdaspur, Pathankot, and Hoshiarpur	Bhatinda, Hoshiarpur
Madhya Pradesh	Bhopal, Chhindwara, and Ratlam	Chhindwara, Ratlam
Maharashtra	Bhandara, Satara, Sindhudurg, and Wardha	Wardha
Telangana	Karimnagar, Jagtial, Rajanna Siricilla, Peddapally, Jayashankar Bhupallapally, Warangal (Urban), Warangal (Rural), Jangaon and Mahabubabad	Jagtial, Jangaon
Kerala	Kannur, Thiruvananthapuram, Thrissur, and Wayanad	Kannur, Thrissur

Supplementary Table 2: Mean age, body mass index, systolic and diastolic blood pressure (average of second and third reading) among all people with hypertension, among those aware of the diagnosis, and newly detected hypertensives in nine districts in India, 2018-19.

	**Hypertension**			**Aware of Hypertension Diagnosis**			**Newly Detected Hypertension**		
	Male (n=2956)	Female (n=4091)	All (n=7047)	Male (n=1264)	Female (n=2427)	All (n=3691)	Male (n=1692)	Female (n=1664)	All (n=3356)
	Mean (SD)	Mean (SD)	Mean (SD)	Mean (SD)	Mean (SD)	Mean (SD)	Mean (SD)	Mean (SD)	Mean (SD)
Age	48 (13)	53 (11)	51 (12)	53 (11)	55 (10)	54 (11)	45 (13)	50 (12)	47 (13)
Systolic blood pressure (mmHg)	152 (18)	152 (19)	152 (19)	151 (21)	150 (21)	150 (21)	153 (15)	155 (16)	154 (15)
Diastolic blood pressure (mmHg)	92 (11)	87 (11)	90 (12)	90 (13)	86 (12)	87 (12)	94 (10)	90 (10)	92 (10)
Body mass index (kg/m2)	25.1 (4.6)	26.0 (5.2)	25.6 (5.0)	25.7 (4.6)	26.7 (5.2)	26.4 (5.0)	24.6 (4.5)	25.0 (5.1)	24.8 (4.8)

Supplementary Table 3: Prevalence, awareness, treatment, and control of hypertension by age groups and gender in nine districts in India, 2018-19.

State - District		Prevalence		Aware		Treatment		Control		Treatment among aware		Control among aware	
	N	n	% (95% CI)	n	% (95% CI)	n	% (95% CI)	n	% (95% CI)	n	% (95% CI)	n	% (95% CI)
**Punjab - Hoshiarpur**													
Male	1178	442	37.5 [34.8,40.3]	185	41.9 [37.3,46.5]	147	33.3 [29.0,37.8]	26	5.9 [4.0,8.5]	147	79.5 [73.0,84.7]	26	14.1 [9.8,19.8]
Female	1970	591	30.0 [28.0,32.1]	419	70.9 [67.1,74.4]	378	64.0 [60.0,67.7]	122	20.6 [17.6,24.1]	378	90.2 [87.0,92.7]	122	29.1 [25.0,33.7]
<45 years	1886	350	18.6 [16.9,20.4]	164	46.9 [41.7,52.1]	135	38.6 [33.6,43.8]	54	15.4 [12.0,19.6]	135	82.3 [75.7,87.4]	54	32.9 [26.2,40.5]
>=45 years	1262	683	54.1 [51.4,56.9]	440	64.4 [60.8,67.9]	390	57.1 [53.4,60.8]	94	13.8 [11.4,16.6]	390	88.6 [85.3,91.3]	94	21.4 [17.8,25.4]
All	3148	1033	32.8 [31.2,34.5]	604	58.5 [55.4,61.4]	525	50.8 [47.8,53.9]	148	14.3 [12.3,16.6]	525	86.9 [84.0,89.4]	148	24.5 [21.2,28.1]
**Punjab - Bathinda**													
Male	1280	403	31.5 [29.0,34.1]	170	42.2 [37.5,47.1]	103	25.6 [21.5,30.0]	15	3.7 [2.3,6.1]	103	60.6 [53.1,67.7]	15	8.8 [5.4,14.1]
Female	1854	476	25.7 [23.7,27.7]	302	63.4 [59.0,67.7]	219	46.0 [41.6,50.5]	52	10.9 [8.4,14.1]	219	72.5 [67.2,77.3]	52	17.2 [13.4,21.9]
<45 years	2061	370	18.0 [16.4,19.7]	152	41.1 [36.2,46.2]	84	22.7 [18.7,27.3]	22	6.0 [4.0,8.9]	84	55.3 [47.3,63.0]	22	14.5 [9.7,21.0]
>=45 years	1073	509	47.4 [44.5,50.4]	320	62.9 [58.6,67.0]	238	46.8 [42.5,51.1]	45	8.8 [6.7,11.6]	238	74.4 [69.3,78.9]	45	14.1 [10.7,18.3]
All	3134	879	28.0 [26.5,29.6]	472	53.7 [50.4,57.0]	322	36.6 [33.5,39.9]	67	7.6 [6.0,9.6]	322	68.2 [63.9,72.3]	67	14.2 [11.3,17.6]
**Madhya Pradesh - Ratlam**													
Male	1543	347	22.5 [20.5,24.6]	75	21.6 [17.6,26.3]	40	11.5 [8.6,15.3]	11	3.2 [1.8,5.6]	40	53.3 [42.1,64.3]	11	14.7 [8.3,24.6]
Female	1864	324	17.4 [15.7,19.2]	116	35.8 [30.8,41.2]	75	23.1 [18.9,28.1]	19	5.9[3.8,9.0]	75	64.7 [55.6,72.8]	19	16.4 [10.7,24.3]
<45 years	2221	257	11.6 [10.3,13.0]	39	15.2 [11.3,20.1]	20	7.8 [5.1,11.8]	6	2.3 [1.1,5.1]	20	51.3 [36.0,66.4]	6	15.4 [7.1,30.3]
>=45 years	1186	414	34.9 [32.2,37.7]	152	36.7 [32.2,41.5]	95	22.9 [19.1,27.2]	24	5.8 [3.9,8.5]	95	62.5 [54.5,69.8]	24	15.8 [10.8,22.5]
All	3407	671	19.7 [18.4,21.1]	191	28.5 [25.2,32.0]	115	17.1 [14.5,20.2]	30	4.5 [3.1,6.3]	115	60.2 [53.1,66.9]	30	15.7 [11.2,21.6]
**Madhya Pradesh - Chhindwara**													
Male	1336	314	23.5 [21.3,25.9]	53	16.9 [13.1,21.4]	37	11.8 [8.7,15.8]	12	3.8 [2.2,6.6]	37	69.8 [56.3,80.6]	12	22.6 [13.3,35.8]
Female	1809	430	23.8 [21.9,25.8]	110	25.6 [21.7,29.9]	98	22.8 [19.1,27.0]	25	5.8 [4.0,8.5]	98	89.1 [81.8,93.7]	25	22.7 [15.8,31.5]

<45 years	2052	273	13.3 [11.9,14.8]	31	11.4 [8.1,15.7]	23	8.4 [5.7,12.4]	7	2.6 [1.2,5.3]	23	74.2 [56.2,86.5]	7	22.6 [11.2,40.4]
>=45 years	1093	471	43.1 [40.2,46.0]	132	28.0 [24.2,32.3]	112	23.8 [20.1,27.8]	30	6.4 [4.5,9.0]	112	84.8 [77.7,90.0]	30	22.7 [16.4,30.7]
All	3145	744	23.7 [22.2,25.2]	163	21.9 [19.1,25.0]	135	18.1 [15.5,21.1]	37	5.0 [3.6,6.8]	135	82.8 [76.2,87.9]	37	22.7 [16.9,29.8]
**Maharastra - Wardha**													
Male	1573	302	19.2 [17.3,21.2]	104	34.4 [29.3,40.0]	84	27.8 [23.1,33.1]	46	15.2 [11.6,19.7]	84	80.8 [72.1,87.2]	46	44.2 [35.0,53.9]
Female	1846	323	17.5 [15.8,19.3]	167	51.7 [46.3,57.1]	139	43.0 [37.7,48.5]	63	19.5 [15.5,24.2]	139	83.2 [76.8,88.2]	63	37.7 [30.7,45.3]
<45 years	2000	183	9.2 [8.0,10.5]	42	23.0 [17.4,29.6]	29	15.8 [11.2,21.9]	11	6.0 [3.4,10.5]	29	69.0 [53.7,81.1]	11	26.2 [15.1,41.4]
>=45 years	1419	442	31.1 [28.8,33.6]	229	51.8 [47.1,56.4]	194	43.9 [39.3,48.6]	98	22.2 [18.5,26.3]	194	84.7 [79.5,88.8]	98	42.8 [36.5,49.3]
All	3419	625	18.3 [17.0,19.6]	271	43.4 [39.5,47.3]	223	35.7 [32.0,39.5]	109	17.4 [14.7,20.6]	223	82.3 [77.3,86.4]	109	40.2 [34.5,46.2]
**Telangana - Jagtial**													
Male	1127	269	23.9 [21.5,26.4]	155	57.6 [51.6,63.4]	122	45.4 [39.5,51.3]	52	19.3 [15.0,24.5]	122	78.7 [71.6,84.5]	52	33.5 [26.6,41.3]
Female	2226	404	18.1 [16.6,19.8]	262	64.9 [60.1,69.4]	189	46.8 [42.0,51.7]	93	23.0 [19.2,27.4]	189	72.1 [66.4,77.2]	93	35.5 [29.9,41.5]
<45 years	2029	162	8.0 [6.9,9.3]	67	41.4 [34.0,49.1]	45	27.8 [21.4,35.2]	20	12.3 [8.1,18.4]	45	67.2 [55.1,77.3]	20	29.9 [20.1,41.8]
>=45 years	1324	511	38.6 [36.0,41.2]	350	68.5 [64.3,72.4]	266	52.1 [47.7,56.4]	125	24.5 [20.9,28.4]	266	76.0 [71.2,80.2]	125	35.7 [30.9,40.9]
All	3353	673	20.1 [18.7,21.5]	417	62.0 [58.2,65.6]	311	46.2 [42.5,50.0]	145	21.5 [18.6,24.8]	311	74.6 [70.2,78.5]	145	34.8 [30.3,39.5]
**Telangana - Jangaon**													
Male	1197	298	24.9 [22.5,27.4]	145	48.7 [43.0,54.3]	111	37.2 [31.9,42.9]	38	12.8 [9.4,17.0]	111	76.6 [69.0,82.7]	38	26.2 [19.7,34.0]
Female	1981	481	24.3 [22.4,26.2]	298	62.0 [57.5,66.2]	232	48.2 [43.8,52.7]	92	19.1 [15.9,22.9]	232	77.9 [72.8,82.2]	92	30.9 [25.9,36.4]
<45 years	1606	166	10.3 [8.9,11.9]	65	39.2 [32.0,46.8]	34	20.5 [15.0,27.3]	13	7.8 [4.6,13.0]	34	52.3 [40.3,64.1]	13	20.0 [12.0,31.5]
>=45 years	1572	613	39.0 [36.6,41.4]	378	61.7 [57.7,65.4]	309	50.4 [46.5,54.4]	117	19.1 [16.2,22.4]	309	81.7 [77.5,85.3]	117	31.0 [26.5,35.8]
All	3178	779	24.5 [23.0,26.0]	443	56.9 [53.4,60.3]	343	44.0 [40.6,47.5]	130	16.7 [14.2,19.5]	343	77.4 [73.3,81.1]	130	29.3 [25.3,33.8]

**Kerala - Kannur**													
Male	957	227	23.7 [21.1,26.5]	143	63.0 [56.5,69.0]	107	47.1 [40.7,53.6]	43	18.9 [14.4,24.6]	107	74.8 [67.1,81.3]	43	30.1 [23.1,38.1]
Female	2302	555	24.1 [22.4,25.9]	388	69.9 [66.0,73.6]	321	57.8 [53.7,61.9]	121	21.8 [18.6,25.4]	321	82.7 [78.6,86.2]	121	31.2 [26.8,36.0]
<45 years	1580	93	5.9 [4.8,7.2]	46	49.5 [39.5,59.5]	26	28.0 [19.8,37.9]	10	10.8 [5.9,18.8]	26	56.5 [42.0,70.0]	10	21.7 [12.1,35.9]
>=45 years	1679	689	41.0 [38.7,43.4]	485	70.4 [66.9,73.7]	402	58.3 [54.6,62.0]	154	22.4 [19.4,25.6]	402	82.9 [79.3,86.0]	154	31.8 [27.8,36.0]
All	3259	782	24.0 [22.6,25.5]	531	67.9 [64.5,71.1]	428	54.7 [51.2,58.2]	164	21.0 [18.3,24.0]	428	80.6 [77.0,83.7]	164	30.9 [27.1,34.9]
**Kerala - Thrissur**													
Male	1222	354	29.0 [26.5,31.6]	234	66.1 [61.0,70.8]	173	48.9 [43.7,54.1]	60	16.9 [13.4,21.2]	173	73.9 [67.9,79.2]	60	25.6 [20.5,31.6]
Female	1962	507	25.8 [24.0,27.8]	365	72.0 [67.9,75.7]	298	58.8 [54.4,63.0]	133	26.2 [22.6,30.2]	298	81.6 [77.3,85.3]	133	36.4 [31.7,41.5]
<45 years	1521	124	8.2 [6.9,9.6]	42	33.9 [26.1,42.6]	27	21.8 [15.4,29.9]	17	13.7 [8.7,21.0]	27	64.3 [48.9,77.2]	17	40.5 [26.9,55.7]
>=45 years	1663	737	44.3 [41.9,46.7]	557	75.6 [72.3,78.5]	444	60.2 [56.7,63.7]	176	23.9 [20.9,27.1]	444	79.7 [76.2,82.9]	176	31.6 [27.9,35.6]
All	3184	861	27.0 [25.5,28.6]	599	69.6 [66.4,72.6]	471	54.7 [51.4,58.0]	193	22.4 [19.8,25.3]	471	78.6 [75.2,81.7]	193	32.2 [28.6,36.1]
**All-District**													
Male	11413	2956	25.9 [25.1,26.7]	1264	42.8 [41.0,44.6]	924	31.3 [29.6,33.0]	303	10.3 [9.2,11.4]	924	73.1 [70.6,75.5]	303	24.0 [21.7,26.4]
Female	17814	4091	23.0 [22.4,23.6]	2427	59.3 [57.8,60.8]	1949	47.6 [46.1,49.2]	720	17.6 [16.5,18.8]	1949	80.3 [78.7,81.8]	720	29.7 [27.9,31.5]
<45 years	16956	1978	11.7 [11.2,12.2]	648	32.8 [30.7,34.9]	423	21.4 [19.6,23.2]	160	8.1 [7.0,9.4]	423	65.3 [61.5,68.8]	160	24.7 [21.5,28.2]
>=45 years	12271	5069	41.3 [40.4,42.2]	3043	60.0 [58.7,61.4]	2450	48.3 [47.0,49.7]	863	17.0 [16.0,18.1]	2450	80.5 [79.1,81.9]	863	28.4 [26.8,30.0]
**All**	29227	7047	24.1 [23.6,24.6]	3691	52.4 [51.2,53.5]	2873	40.8 [39.6,41.9]	1023	14.5[13.7,15.4]	2873	77.8 [76.5,79.1]	1023	27.7 [26.3,29.2]

Supplementary Table 4: Mean difference between first and second reading, first and third reading and second and third reading for systolic and diastolic blood pressure for community survey in nine districts in India, 2018-19.

**BP Readings (n = 7047)**	**First** Mean (SD)	**Second** Mean (SD)	**Diff (95% CI)**	**First** Mean (SD)	**Third** Mean (SD)	**Diff (95% CI)**	**Second** Mean (SD)	**Third** Mean (SD)	**Diff (95% CI)**
Systolic blood pressure (mmHg)	155 (20)	152 (20)	3.1 (2.8 - 3.3)	155 (20)	152 (19)	3.7 (3.5 - 4.0)	152 (20)	152 (19)	0.7 (0.4 - 0.9)
Diastolic blood pressure (mmHg)	90 (12)	89 (12)	0.9 (0.8 - 1.1)	90 (12)	90 (12)	0.6 (0.4 - 0.8)	89 (12)	90 (12)	-0.3 (-0.5 - -0.2)
